# Reach, Recruitment, Dose, and Intervention Fidelity of the GoActive School-Based Physical Activity Intervention in the UK: A Mixed-Methods Process Evaluation

**DOI:** 10.3390/children7110231

**Published:** 2020-11-17

**Authors:** Stephanie T. Jong, Caroline H. D. Croxson, Campbell Foubister, Helen Elizabeth Brown, Cornelia Guell, Emma R. Lawlor, Emma K. Wells, Paul O. Wilkinson, Edward C. F. Wilson, Esther M. F. van Sluijs, Kirsten Corder

**Affiliations:** 1 UKCRC Centre for Diet and Activity Research (CEDAR) and MRC Epidemiology Unit, University of Cambridge, Cambridge CB2 0QQ, UK; s.jong@uea.ac.uk (S.T.J.); cf469@medschl.cam.ac.uk (C.F.); helen.brown@bi.team (H.E.B.); emma.lawlor@mrc-epid.cam.ac.uk (E.R.L.); emma.wells@mrc-epid.cam.ac.uk (E.K.W.); drkirstencorder@gmail.com (K.C.); 2 Faculty of Medicine and Health Sciences, University of East Anglia, Norwich NR4 7TJ, UK; 3 Nuffield Department of Primary Care Health Sciences, University of Oxford, Oxford OX2 6GG, UK; caroline.croxson@hmc.ox.ac.uk; 4 European Centre for Environment and Human Health, University of Exeter Medical School, Knowledge Spa, Royal Cornwall Hospital, Truro TR1 3HD, UK; C.Guell@exeter.ac.uk; 5 Department of Psychiatry, University of Cambridge, Cambridge CB2 8AH, UK; pow12@cam.ac.uk; 6 Cambridgeshire and Peterborough NHS Foundation Trust, Cambridge CB21 5EF, UK; 7 Health Economics Group, Norwich Medical School, University of East Anglia, Norwich NR4 7TJ, UK; Ed.Wilson@uea.ac.uk

**Keywords:** school-based intervention, process evaluation, fidelity, mixed-methods, physical activity

## Abstract

School-based multi-component physical activity (PA) promotion is advocated; however, research has indicated that a multi-component approach may not always be effective at increasing adolescent PA. Evaluation of the GoActive 12-week multi-component school-based intervention showed no effect on adolescent PA. A mixed-methods process evaluation was embedded to facilitate greater understanding of the results, to elicit subgroup perceptions, and to provide insight into contextual factors influencing intervention implementation. This paper presents the reach, recruitment, dose, and fidelity of GoActive, and identifies challenges to implementation. The process evaluation employed questionnaires (1543 Year 9s), individual interviews (16 Year 9s; 7 facilitators; 9 contact teachers), focus groups (48 Year 9s; 58 mentors), alongside GoActive website analytics and researcher observations. GoActive sessions reached 39.4% of Year 9s. Intervention satisfaction was relatively high for mentors (87.3%) and facilitators (85.7%), but lower for Year 9s (59.5%) and teachers (50%). Intervention fidelity was mixed within and between schools. Mentorship was the most implemented component. Factors potentially contributing to low implementation included ambiguity of the roles subgroups played within intervention delivery, Year 9 engagement, institutional support, and further school-level constraints. Multiple challenges and varying contextual considerations hindered the implementation of GoActive in multiple school sites. Methods to overcome contextual challenges to implementation warrant in-depth consideration and innovative approaches.

## 1. Introduction

The health benefits of physical activity are widely demonstrated in the literature [1,2]. For young people in particular, physical activity has been associated with improved mental well-being and a lower risk of obesity [1]. Evidence suggests that adolescent physical activity can have both a direct and indirect positive effect on adult health [3], as physical activity habits track into adulthood [4]. However, the majority of young people in the UK do not meet the current recommendation of at least 60 min/day of moderate to vigorous physical activity (MVPA) [5,6,7]. Furthermore, the literature suggests that the physical activity level declines across the lifespan. UK cohort studies revealed that physical activity declines between the age of five and nine years [8,9]. In adolescence, physical activity declines, on average, 7% per year [10].

In an effort to increase physical activity among young people, researchers have developed and tested various physical activity interventions, many of which have been implemented in schools. Schools are seen as a powerful site to promote physical activity programmes and interventions [11] due to the ability to reach the entire adolescent population. Schools also provide opportunities throughout the school day (including before and after) to positively change behaviour [6], while existing structures within the school environment (e.g., social networks, educator-student relationships, school policies and processes) can be leveraged to integrate physical activity promotion efforts and embed activities into the existing school system. A synthesis of reviews of trials designed to improve physical activity or fitness in adolescents in the school environment reported a positive effect on in-school, out-of-school or overall physical activity [7]. Although the review found a small positive impact on physical activity, a clear picture of effective strategies to increase physical activity in youth was absent.

Capitalizing on the school site, the Get others Active (GoActive) study [12] evaluated the effectiveness of a 12-week multicomponent school-based intervention designed to increase physical activity in 13–14-year-old adolescents (Year 9). GoActive consisted of four essential elements: GoActive in-class sessions, older year group mentors, in-class Year 9 peer leaders, and the GoActive website (including points, school graphs, and claiming rewards). Underpinned by elements of Self-Determination Theory [13], GoActive was developed to improve physical activity through strengthened peer and mentor support, self-efficacy, group cohesion, self-esteem and friendship quality using a tiered-leadership system within schools. The effectiveness evaluation showed no effect on minutes of MVPA at 10-month follow-up [14].

Previous research on school-based physical activity interventions highlighted the need to critically examine the delivery of each component and the processes of any complex intervention [15]. Process evaluation provides detailed evaluative information about the delivery of an intervention with the aim to contextualise and interpret its potential effects, providing greater confidence in conclusions about effectiveness [16]. Additionally, process evaluation facilitates a deeper interpretation of findings and provides greater insight into contextual factors that influence how an intervention works [17], and how an intervention may be applied in other contexts, or to other populations [16]. Emerging school-based physical activity intervention process evaluation research has demonstrated contextual and study implications that have impacted intervention implementation. For example, school-level constraints [18], attendance, engagement, and enthusiasm of participants and facilitators [19,20,21], flexibility and adaptability of the programme [22], the provision of more in-depth training [20], and the need for the provision of greater guidance to teachers throughout an intervention [18]. This shows that process evaluation is an essential aspect of the design and testing.

For GoActive, previous analysis of Year 9 participant satisfaction found that some intervention components were liked, for example, mentorship, but implementation issues undesirably impacted satisfaction [23]. Additionally, the competition component was disliked by girls and shy/inactive students, compared to their male counterparts. The current paper builds on these results by describing intervention reach, recruitment, dose, and fidelity, as well as the challenges to the implementation of GoActive. These process evaluation components allow for the exploration of the complexities of school-based physical activity interventions, and will highlight contextual factors affecting quality implementation.

## 2. Materials and Methods

Ethical approval for the process evaluation was obtained from the University of Cambridge Psychology Research Ethics Committee (PRE.2015.126). All adult participants provided written informed consent, while children (under 16 years old) gave written informed assent and their parents provided passive consent.

### 2.1. The GoActive Intervention

The GoActive intervention, described in detail elsewhere [12,24,25,26], consisted of four essential elements: GoActive in-class sessions, older year group mentors, in-class Year 9 peer leaders, and the GoActive website (including points, and claiming rewards). Each are detailed in Table 1.

### 2.2. Trial Design

The trial methods were published in the study protocol [12], as well as a report of the outcomes [14]. Briefly, the trial was a two-armed, cluster randomised control trial with 16 schools (8 intervention, 8 control). All state-run secondary schools in Cambridgeshire and Essex were eligible for inclusion. All Year 9 students, aged 13–14 years, were invited to take part in the programme evaluation. Data were collected at baseline (T1), mid-intervention (six weeks post-baseline) (T2), and 12–14 weeks post-baseline (T3) and 10 months post-intervention (T4). After baseline measurements, schools were computer-randomised to the GoActive intervention or a no-intervention control condition. Randomisation was stratified by school-level pupil premium (below/above county-specific median) and county (Cambridgeshire/Essex). Pupil premium funding, used as a proxy for school-level deprivation, is school funding that aims to reduce the effects of deprivation [27]. The protocol of the mixed-methods process evaluation has been published elsewhere [26], and only the methods relevant to the analyses presented here will be described in detail below.

### 2.3. Process Evaluation Data Collection

Details of all aspects of the mixed-methods data collected and the levels of response can be found in Table 2. Data collection methods included participant questionnaires, observations, purposively sampled semi-structured individual and focus group interviews, and website analytics.

Direct observations of two GoActive sessions per school were arranged by contact teachers. Teachers were informed of when observations would take place, as well as most mentors. Data collected during observations comprised detailed notes describing what took place, where in the school setting, any informal conversation, the observed role of the mentor, teacher actions, and level of engagement from Year 9s.

Website analytics included individual points logged, rewards claimed, activities selected, and messages from mentors. All mentors and facilitators were asked to complete intervention delivery logs via the GoActive website. These logs sought information on, for example, the date each lesson took place, how long it lasted, how many Year 9s were active in their participation, and any comments about the delivery of the session.

**Figure 1:**
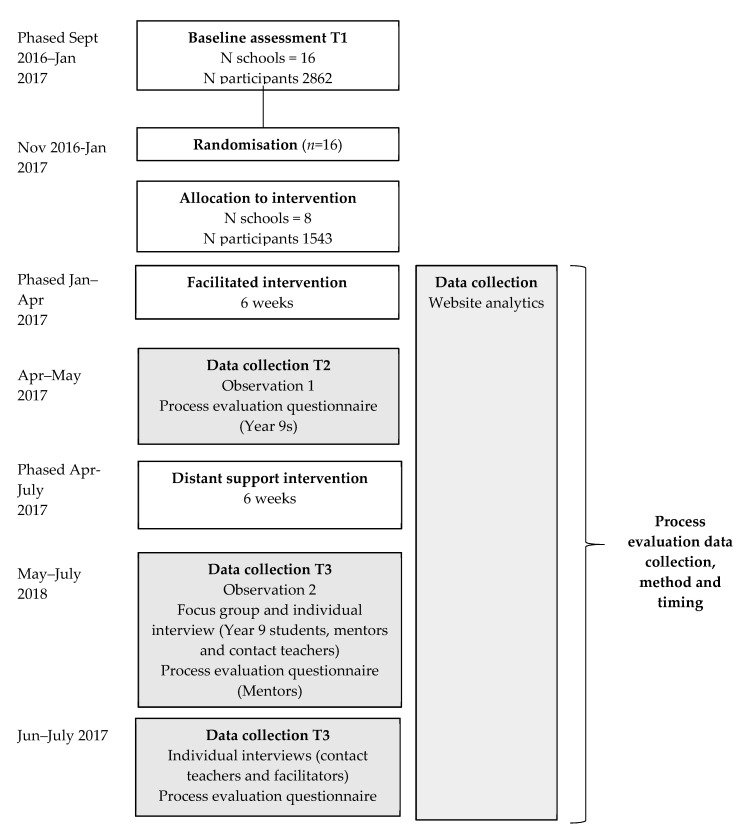
Process evaluation data collection method and timing. (Grey boxes denote data collection periods; T = time point).

Here, we present process evaluation data collected at mid-intervention (six weeks post-baseline, T2) and post-intervention (12–14 weeks post-baseline; T3) [26]. Anecdotal reports suggested there were delays in intervention delivery at T2; therefore, most of the data from this paper pertain to T3, unless specifically stated otherwise. The timing of the quantitative and qualitative data collection was concurrent. Figure 1 shows the intervention, and timings of the process evaluation data collection methods. Data on the process evaluation measures were collected to evaluate whether findings were consistent with how the intervention was theorised to act in the GoActive logic model [26], and any potential barriers to wider dissemination should the programme prove effective.

### 2.4. Sampling

The T2 questionnaire allowed Year 9 students at intervention schools to indicate whether they would be happy to be contacted about taking part in an interview. Year 9 students who responded positively were provided with an additional information sheet to clarify the interview procedure (both individual and focus group).

The whole-year level approach of GoActive aimed to avoid stigmatisation of targeting particular groups. The development of the intervention involved listening to the voices of people with characteristics that were deemed to be common in individuals who were hard to reach in physical activity interventions, including girls and individuals with high levels of shyness and inactivity [25]. Participants included in our process evaluation were purposively sampled to account for perspectives of individuals with these characteristics. Two shy and inactive individuals per school were invited to participate in an individual interview. Individuals were purposely sampled based on T1 (baseline) questionnaire data. The interview selection strategy aimed to provide a greater understanding to researchers of how to better target populations most in need of health promotion. Shyness and sociability data were provided by two 5-item measures from EAS temperament scale [28], included in the T1 questionnaire data. Physical activity was determined by baseline self-reported youth physical activity questionnaire (YPAQ) data. Students who exhibited greater degrees of shyness and sociability (lowest scoring tertile) and those who participated in the least physical activity (lowest scoring tertile) were invited to interview. Of the students who consented to being interviewed, two Year 9s were randomly selected per school. In some schools, selected students were vetted by the contact teacher. An additional option was provided to schools if they did not approve the initial random selection. A one-to-one interview was proposed to be more comfortable for these individuals.

Focus group participants were grouped based on tertiles of website usage as a proxy for intervention engagement (150 points (high), 10–100 points (medium), ≤  10 points (low)), and purposively sampled to aim for a mix of sexes, with participants from a variety of tutor groups.

All mentors, contact teachers, and facilitators were invited to interview. All teachers were provided with questionnaires via the contact teacher at the school. The number of participants and data collection methods are provided below and summarised in Table 2.

### 2.5. Analysis

#### 2.5.1. Data Preparation

Quantitative process evaluation data were extracted from the student questionnaires, teacher questionnaires, and facilitator questionnaires. Website analytics were extracted from the GoActive website database more than a year after active intervention delivery had ceased (October, 2018). Website analytics were recorded in Microsoft Excel and imported into Stata [29] for processing and analysis.

Qualitative data from individual and focus group interviews were voice recorded and transcribed verbatim. Data from the observations were originally recorded in a notebook, and typed into a narrative using Microsoft Word on the day of observation.

#### 2.5.2. Data Analysis

Qualitative and quantitative data were analysed separately, producing two sets of findings, and mixed during interpretation. Descriptive summary statistics (means or medians, standard deviations or interquartile ranges and/or percentages) were calculated using an intention to treat approach for a number of quantitative variables.

All interview data were analysed by the process evaluation lead in NVivo 12 [30], using a six-step thematic approach [31]. The coding schemes emerged through both inductive and deductive approaches generated from the topics in the interview guides, as well as iteratively from the data. All codes were discussed with a second coder, and were categorised as a series of themes. The themes were discussed, refined, and agreed by both researchers. The themes illustrated in this paper exemplify the process evaluation components. These themes combine with an inductive analysis to grasp the challenges of intervention delivery in the complex school system. Illustrative anonymised quotes typify the data from interviews. Observation qualitative data were used to provide context, or support, reaffirm, or contradict data from interviews, and were documented as text extracts.

Given the project’s multiple datasets and the need to generate an integrated set of findings, two researchers worked together to compare and integrate findings from the datasets. Initially, the findings were sorted based on the process evaluation components: reach, recruitment, dose delivered (completeness), dose received (exposure), fidelity, and dose received (satisfaction) (Table 3). Findings were reviewed and compared to assess convergence, and dissonance between the datasets, and specific examples of qualitative data were gathered to reflect convergence or dissonance, or to explain particular process evaluation components. The researchers clarified interpretations of the findings where required. Results were discussed with the research team for review and clarification.

## 3. Results

Descriptive findings of quantitative assessments of reach, recruitment, dose delivered (completeness), dose received (exposure), fidelity, and dose received (satisfaction) findings are presented. Qualitative data provide a nuanced picture to contextualise the questionnaire data, and will be presented in the section below, alongside key quantitative results. The final section of the results will focus on the perceived challenges to intervention implementation.

### 3.1. Reach

The reach, or proportion of participants who attended at least one GoActive session during tutor times, calculated from a self-reported Year 9 student questionnaire during the distant support phase, was 39.4%.

### 3.2. Recruitment

Procedures to attract and maintain participant involvement in the intervention included rewards. On a 5-point Likert scale ranging from “Do not like it at all” (1) to “Like it a lot” (5), 38.3% of Year 9 students reported liking rewards (mean of 3.8 (standard deviation (SD) 1.4)). Additional ‘thank you’ gifts were provided after each measurement session. These included pens, earphones, mints, and stationery, which may have facilitated sustained involvement. In turn, 87% of Year 9 students were retained at mid-intervention (T2), 80% post-intervention (T3), and 76% at 10 months post-intervention (T4).

The top three reasons for mentors joining the programme reported from the questionnaire data were (1) the incentives/prizes (£20 vouchers and a hoodie), (2) to be more active themselves, and (3) because a teacher encouraged them to. In the focus group discussions, mentors suggested that their continued involvement was linked to ‘fun’ and enjoyment, or the social aspect of spending time with their peers. Others stated that they believed in the aims of GoActive, and that their involvement was linked to perceptions that GoActive was ‘good for their (Year 9s) health’.

### 3.3. Dose Delivered (Completeness) and Dose Received (Exposure)

The dose delivered (completeness), or the number of GoActive intervention components implemented, differed between and within schools. This was typified in interviews across Year 9s and mentors, in particular in relation to in-class peer leaders and the ability to log website points. Focus groups with subgroups explained that changes in the delivery of GoActive sessions in particular was in response to competing school priorities, which impacted resourcing, such as school space availability, a lack of time, or engagement issues. One contact teacher explained:

*When it’s exam season it’s exam season for [Year] 9, 10 and 11, so we had to stop for a certain point… we had to stop because of the sports hall and gym were being used and we couldn’t get the kids out during registration because their exams started at nine o’clock*.

(Contact teacher, School H)

The complete GoActive programme was not implemented by any school. Table 4 depicts the implementation of the four GoActive essential components per school reported by Year 9 students, website analytics, and number of website logs completed by mentors and facilitators.

The reported dose received (exposure) of engaging with at least one GoActive class session in the last two weeks during the distant support phase ranged from 11.2% to 63.2% between schools, as reported by Year 9s (Table 4). This contrasted with the number of sessions being delivered; 93.7% of the mentors and 84.2% of the teachers reported that the sessions had been delivered at least once over the last two weeks in this phase.

Interview data indicated that the dose received (exposure) changed throughout the intervention, primarily linked to difficulties implementing the GoActive sessions consistently. Engagement with GoActive sessions varied per school, with many mentors describing boys as being more likely to engage and interact within the GoActive sessions. However, there were a number of factors that impacted student engagement. As one example, student behaviour was a constant factor that impacted others’ participation in the sessions. One mentor described (School H):

*A lot of the boys just like didn’t co-operate very well and they were just trying to be, like, silly with their friends and weren’t bothered.*

This sentiment around behaviour and disengagement was reiterated across all mentor focus groups, and in interviews with facilitators, who articulated the challenges presented to them from mentors.

### 3.4. Intervention Fidelity

From the data presented in Table 4, the fidelity, or the extent to which the GoActive intervention was implemented as planned, varied by school. It should be noted that despite reporting that no similar programmes were running at the school pre-intervention, interviews with Year 9 students, mentors and the contact teacher revealed that School D had been running a weekly ‘Healthy Active form time’ activity session with all year groups in the school.

Each of the four GoActive essential elements (Table 1) will be discussed in relation to intervention fidelity. Table 5 compares GoActive protocol with school implementation for each GoActive tenet: novelty, choice, flexibility, competition, mentorship, and rewards.

#### 3.4.1. GoActive Sessions:

Qualitative data from individual and focus group interviews indicated that most schools attempted to implement the GoActive sessions as planned. Most descriptions of the GoActive sessions included the class going to a location within the school (e.g., AstroTurf, hall, field etc.), and mentors facilitating an activity session. One Year 9 participant described:

*We normally like go into form and we get told where to go and then we meet the mentors where we were supposed to be.*

(Year 9 focus group, School C)

A Year 9 participant from School E describes a similar process:

*We usually just go on the field and do like rounders, football, any sport on the GoActive website, and just go on the field and do it as a form.*

(Year 9 focus group, School E)

The time for GoActive varied between schools due to contextual school differences in timetabling. Most schools used their form time (registration/tutor time) at the beginning of the day, which varied from 15 to 25 min. One school used their afternoon form time, and another school implemented after school sessions in line with their after-school clubs.

Quantitative and qualitative data on GoActive sessions present dissonance between datasets. As one example, 55.4% of Year 9s from School G reported receiving at least one GoActive session in the last two weeks (Table 4). However, data from individual interviews with Year 9 students, and the two observations, raised questions as to whether GoActive had been implemented at the school at all. During one observation, anecdotal comments from Year 9 students revealed that the GoActive session was a ‘one off’ session run for the purpose of the observation. Other indications from the day of observation, including teacher’s comments, led to further questions around implementation. The following is an excerpt from the second observation:

*As we packed up the equipment and walked over to the gate I met one of the form tutors. In the absence of older mentors, I asked, ‘Do you have any older mentors helping to run GoActive?’ She replied ‘No’.*

On observation, many schools implemented the GoActive sessions as intended in the design of the intervention (Table 1); however, some modifications were present. From the interviews and observations, modifications to the GoActive sessions included:

- The role of the mentor: some mentors were given greater responsibility to organise, run and facilitate the delivery of the session than others. Some teachers employed greater control over the organisation and delivery of the session than others.
- An omission of the mentor role
- Combined class GoActive sessions
- Gender segregated GoActive sessions
- Non-GoActive activities selected for GoActive sessions
- Separate activities for those who did not want to participate in the main session, e.g., some Year 9s were asked to walk around the playing fields if they did not want to participate in the main activity

School B amended the GoActive sessions to account for vertical forms (where the form/tutor group is made up of students from all year groups, as opposed to a single year group). Instead of conducting an activity with the whole form, the mentors recruited Year 9s via a sign-up sheet to different GoActive activities run throughout the week. Year 9s were required to sign up, remember the date of the session, and turn up at the time and place where it was held, before participating. In turn, this led to some Year 9s reporting that ‘I wasn’t really asked’ to participate in the session, and as such, they did not participate. Quantitative results offer a complimentary perspective as Year 9 participants at School B reported the fewest class sessions (11.9%) (Table 4). Despite having traditional form groups, School F employed a similar mechanism of recruitment to GoActive sessions, trialling after school sessions. School F also reported low GoActive class sessions (20.8%).

Noteworthy, some Year 9 students described that they did not receive any GoActive sessions. One participant stated, ‘We didn’t do anything’ (Year 9 focus group discussion, School H). This is reflected in the quantitative data, with only 13.7% of Year 9s at School H reporting participating in a GoActive session in the last two weeks when schools were still meant to be running GoActive (Table 4).

#### 3.4.2. Mentors

Data from the observations, and Year 9 and mentor focus group interviews revealed that each tutor group had between two and seven mentors (Table 4). Mentor age ranged from Year 10 students (14–15 years old) through to Year 13 students (17–18 years old), used within one school. Some mentors demonstrated their engagement with the GoActive programme by discussing their pre-GoActive session plans, working with the Year 9 students in multiple tutor times, demonstrating how to play, joining in, and working with Year 9 students to encourage their participation in GoActive sessions. In an observation at School A, one mentor demonstrated their engagement:

*The mentor walked over to the boy who stood in the corner and gave him a ball. He looked to encourage the boy to participate, and urged the boy to throw the ball at an opposing player. The mentor moved away and the boy moved forward to throw it.*

Observational data revealed diverse actions of the mentors between schools. The following is an excerpt from an observation at School D:

*We walked over to the sport shed. The contact teacher had given their keys to a Year 10 mentor in order for them to gather the equipment they needed. The two mentors were throwing a Frisbee between themselves. They did not appear to speak to any Year 9 students, nor give any eye contact to anyone. They purely played between themselves.*

Interview data from Year 9 students further depicted the disparity of the implementation of the mentor role. Some Year 9s reported that they had not seen their mentors ‘for a couple of weeks’, or that they only ‘sometimes turned up’. At some schools, disorganisation was another factor that resulted in the GoActive sessions not being implemented:

*A couple of times they’ve (mentors) shown us the cards [Quick Cards–GoActive resources] with the different selection of activities and we’ll talk about which ones we want to do…but then they don’t book a place to do it or they don’t have a football next time so we don’t end up doing it.*

(Year 9 focus group, School D)

#### 3.4.3. In-Class Year 9 Peer Leaders:

Contact teachers, mentors and facilitators reported that all schools found it difficult to implement the in-class Year 9 peer leader GoActive component. Observations suggested that no schools had implemented in-class Year 9 peer leaders. Reports from Year 9 individual interviews and focus groups, as well as individual interviews with contact teachers, supported this. A contact teacher from School D explained their rationale for taking the focus off in-class Year 9 peer leaders:

*I think we haven’t had any Year 9 Peer Leaders, I think that’s a difficult thing to try and do because it’s difficult to lead your own peer group, and some, it’s something that I would maybe explore further next time. If I’m honest I haven’t invested much time into that aspect of it, I invested more time in the coordination of it and the Year 10 Leaders (mentors) going out. I can see how some peer leaders do encourage and motivate some others, you know, the enthusiastic ones, that might help, but I think it’s, that’s, it’s another thing for teachers or leaders to have to do, who’s going to be the leader next time and rotate it round, it’s just another extra thing which I’m not sure is necessarily needed.*

Conversely, quantitative data from Year 9 questionnaires suggest some implementation of in-class peer leaders at every school, with the lowest implementation in two sessions (Table 4).

#### 3.4.4. GoActive Website Use–Points Logged

Engagement with the recording of points varied by school, for both the percentage of Year 9s who recorded points, and the average points that were claimed (Table 4). Overall, only 714 Year 9s (46.2%) logged points on the GoActive website. Year 9s reported that they were aware of their requirement to log their individual points on the GoActive website in individual and focus group interviews. Year 9s discussed challenges with forgetting their individual profile password to the GoActive website, forgetting to log their points, and subsequently adding numerous points at one time, having to recall activity participation from memory, as well as issues with schools’ resourcing of laptops/computers to facilitate logging points. There was substantial reliability in using form time to log points from Year 9s; very few Year 9s reported logging points outside of school hours:

*… because some people did log at home, like I think I logged on at home once, one week and then between like four weeks I didn’t log my points, I had to log on the fifth week my points from the four weeks.*

(Year 9 focus group, School D)

#### 3.4.5. GoActive Website Use–Claimed Rewards

Year 9 participants from all schools claimed GoActive rewards. However, there was great variation in claimed rewards from participants by school (Table 4). For example, only three prizes were claimed by one participant at School H, whereas 230 prizes were claimed by 106 participants at School E.

In most of the individual and focus group discussions, Year 9s acknowledged the GoActive rewards. There was confusion with who to contact about claiming rewards from the website, and who would distribute the rewards. Year 9s reported disappointment with the time it took to receive the reward after logging points and claiming the reward online. One student reported:

*Ours tried (referring to mentors), they wrote down like who’d received the jumpers and stuff, but then they didn’t give us them one week, the next week they didn’t again, and then the next week they were off on exams so we haven’t actually seen them since then.*

(Year 9 focus group, School E)

There was additional confusion about how the rewards worked generally. For example, some Year 9s did not understand the process of claiming the rewards. A few Year 9s stated that they were saving their points to claim a GoActive hoodie, instead of claiming the other GoActive rewards along the way. In another focus group, one Year 9 asked if they were required to buy the rewards. The other Year 9s in the focus group were able to inform them of the process of acquiring rewards.

### 3.5. Dose Received (Satisfaction): Multi-Subgroup Response to the Intervention

Dose received (satisfaction) was monitored for Year 9s, mentors, teachers and facilitators. Questionnaire data revealed 59.5% of Year 9 students thought that GoActive was fun. Qualitative data were resoundingly positive, revealing that Year 9s found the programme fun, and preferred it to how they traditionally used their form time. Of the mentors, 87.3% thought that participating in the programme was enjoyable. Half of the teachers, 50% (10/20), reported that they enjoyed facilitating GoActive, and 70% (14/20) indicated that they would recommend it to a colleague. The vast majority (85.7%, 6/7) of facilitators indicated that they would recommend the GoActive programme to a colleague.

### 3.6. Challenges to Implementing the GoActive Intervention

Based on observations, and individual and focus group interview data, primary factors that may have contributed to the lack of implementation include: ambiguity of the roles subgroups played within GoActive, Year 9 disengagement, a lack of institutional support for contact teachers, and school level constraints, e.g., uniform requirements, limited facility space, resources and time. Additional school-level constraints negatively impacting implementation included teacher absence, and the timing of the intervention within the school year. These will be discussed in greater detail below.

#### 3.6.1. Ambiguity of GoActive Delivery Roles

One of the key factors contributing to the lack of implementation fidelity appeared to be the uncertainty of the roles that each subgroup played. Most of the contact teachers stated that they were tasked with their role in implementing GoActive by their Senior Leadership Team (SLT), or one of the Heads of Year. A few contact teachers stated that some members of the SLT were extremely supportive and proactive, taking a keen interest in physical activity:

*Because our school are very proactive, the Head Teacher likes the idea of physical activity and our Heads of Year are engaged with it, they are happy for a form time to be used in that manner.*

(Contact Teacher, School D)

Other contact teachers indicated that their SLT did not fully comprehend the intervention. Most of the time, responsibility of the intervention remained solely with this member of staff:

*Initially, I didn’t realise how much was involved and, to be honest, I don’t think my Head knew how much was involved, I think he thought it would be, I don’t think he’d maybe read the information through and he didn’t realise, I think he thought it might be a month or two thing, done, he didn’t realise it was going to go on.*

(Contact Teacher, School A)

This was reinforced by the Contact Teacher at School G:

*I’ve found that that has been the biggest pressure of it, that I may… Because I am the Head of Year by myself, I haven’t got an assistant, I haven’t got anyone else helping me and I’ve found that this has been, not something that, but I just haven’t been able to impart as much of my time on this as maybe I want to but I’m just unable to do that, so again, maybe someone else within the school could have taken it on but they’re so busy, staff are so busy so I would say that’s because of my tutor team and I’m relying on them to be more proactive with it and they’re not.*

At School D, the contact teacher held an SLT role within the school, aligning with health and wellbeing. The contact teacher at School D felt that their position within the school helped to facilitate intervention implementation, and whole year level adoption of GoActive. For example, they were able to use their presence in the team to facilitate staff engagement, and schedule GoActive sessions into their timetable, creating a sense of routine, which was highly valued by Year 9s.

Year 9s held mentors and tutors responsible for the successful implementation of the GoActive programme. The following comment exemplifies this:

*I think our form tutors were relying on the mentors to come and get us but because our mentors didn’t, our form tutors just forgot that we had to do it.*

(Year 9 focus group, School H)

Actions of tutors described by Mentors and Year 9s also indicated that more responsibility was placed on the mentor. Year 9s relied on the mentors to be competent at the GoActive activities, prepare the equipment, explain the rules, interact with those who were not engaging, and continuously encourage those who did not participate. Furthermore, mentors felt that they had to manage the classroom, and Year 9 student behaviour. For some tutor groups, if mentors or tutors did not organise an activity, the GoActive sessions did not take place:

*We tried to (organise) but we’re a bit wimpy and our form tutor doesn’t really want to and then our Year 10 leaders [mentors] aren’t very good so we don’t really get to actually do it.*

(Year 9 focus group, School D)

A Year 9 focus group from School F further reiterated this ambiguity of responsibility:

*I think more on like our side of the school as opposed to the actual project because we just haven’t really done it like done much.*

(Student 1)

*And I don’t think there’s like that many people like in our form for example like willing to take responsibility for setting it up.*

(Student 2)

*Yeah.*

(Student 1)

*Because everyone’s like, oh if I don’t do it we won’t have to do the sport.*

(Student 2)

*Yeah, I think we’re just like relying on our mentors but then if they don’t…*

(Student 1)

#### 3.6.2. Year 9 Student Disengagement

Year 9 student behaviour, attitudes, and disengagement were discussed as a challenge to implementation by mentors and Year 9 students themselves. Age between the year groups, mostly for those mentors who were in Year 10, was constantly cited as an issue for managing attitudes and behaviour within the Year 9 cohort by mentors. Mentors expressed concerns with what they described as a lack of ‘respect’ shown by Year 9 students:

*They wouldn’t listen to us because we’re just students as well. So you’d tell them what to do and then they’d do it for like five minutes and then it’d just turn into like a free-for-all.*

(Mentor focus group, School E)

One contact teacher (School F) stated that mentors ‘complained of apathy’ and, “Oh, they (Year 9s) don’t want to know, they’re not bothered”’. Another contact teacher commented that mentors reported finding it difficult to ‘motivate’ the Year 9s. Despite some mentors working through disengaged behaviours and attitudes, there were still reported difficulties:

*Once we kind of got them involved, we kind of had like a few, like maybe 3 or 4 boys that weren’t involved and they were kind of like swaying the whole class.*

(Mentor focus group, School D)

Facilitators and contact teachers encouraged mentors to promote participation from Year 9 students. Concerns were continually expressed about the lack of Year 9 students engaging with activities. One contact teacher (School D) explained how they tried to reassure the mentors:

*A little bit of worry about what the expectation is as well, so you know, I was sort of saying to them ‘if you decide to do this Zumba session that you did inside and only five people do it, it’s fine, just go with it, you know, you’re not there to make people do it, you’re there to facilitate’, and I think there’s a little bit of a maybe worry with that, like ‘I can’t get everybody to do it’ or an embarrassment maybe as well about being that sort of enthusiastic and then not responding to that maybe, I don’t know.*

At some schools, there was a trade agreed: if Year 9s did not want to participate in the chosen activity, they were permitted to walk around the activity space to attain GoActive points (walking was an activity that could be logged to attain GoActive points). The following is an abstract from an observation from one school:

*A girl approaches one form tutor with a small group of girls and asks ‘Ma’am, do we have to do it?’ with a sad, whining tone. This was followed by a “Yeah” in agreement from her peers. The tutor informs them that they will have to “Ask Sir”, who is running the session. They walk over to the teacher who is surrounded by the group of Year 9 students. He informs them that they can participate in the session, or if they choose not to then they need to walk around the field instead. Some girls leave the group (those who asked the female tutor if they could not participate). A different girl watches the girls leave the group, raises her hand to point towards them, and begins to ask “why is everyone…?” She does not finish her question, but she continues to stare at those walking. She stays involved in the main session. Twelve girls in total decide to walk. Two boys also decide to walk around in the opposite direction.*

#### 3.6.3. School-Level Barriers

##### Uniform not Suitable for Activity Participation

The school uniform was a challenge to Year 9 participation that was mentioned by Year 9s, mentors and contact teachers. Many schools required the Year 9s to change into their Physical Education sporting uniform to participate. At observed sessions, this change took approximately 5–10 min of a 15–25 min form time. At some schools, Year 9s were excluded from participating due to not bringing sport shoes. Some schools allowed Year 9s to wear their traditional school uniform during GoActive sessions. However, this brought other challenges that impacted Year 9 participation, including sweating and smelling in their uniform after participating in a GoActive session, or wearing skirts, which was a key barrier to girls’ participation noted by multiple subgroups:

*Just the fact that girls wear skirts and that can be a bit difficult when you’re like really trying to go for basketball or something, I mean we were just standing around and a ball bounced up a girl’s skirt [laughs], yeah. So I guess it can be embarrassing in that kind of way.*

(Mentor focus group, School D)

An observation at School E further cemented this as an issue for participation for girls:

*Whilst walking back to the boys game the teacher says “it’s like getting blood out of a stone for some of the girls” … “even the sporty ones”. After a brief interaction he indicated that “its ‘cos of their skirts”. He said that even if they did want to run they were in their school skirts, which made the girls feel uncomfortable.*

##### Lack of Resources and Facilities

Another challenge mentioned by a number of schools was the resourcing of facilities. Mentors discussed that they ‘didn’t have the equipment’ they wanted to run the activities. Space was limited by exam provision, but also by other form groups using the space at the same time, or the school not having the space to run activities:

*One of the main frustrations was getting the facilities, because we had like two or three forms all doing dodgeball.*

(Mentor focus group, School E)

Mentors and contact teachers discussed difficulties with booking computer rooms for Year 9s to log points. Schools either lacked the resources, or the computer rooms were used by other form groups during registration time. Mentors also noted that they did not possess the ability to book computer rooms. A contact teacher from School F added that the quality of facilities was also a challenge:

*Our IT facilities aren’t very good at the moment, and if you want the students to log their points, they may not remember at home because maybe they won’t regard it as extra homework or something, I don’t know, or they might just forget to do it…So, if you want to try and encourage them to log their points, it’s best to book like an IT facility during registration time, which we’ve tried, but because our facility’s quite slow and you’ve only got twenty minutes to do it in, by the time you’ve logged on it’s time to log off again.*

##### Competing School Priorities

Exam timings and other school priorities were discussed by contact teachers, Year 9s, and mentors as other key issues that had an impact on implementation. Exam timings created inconsistencies in mentor availability, and where the programme could be run within the school. For some, these challenges contributed to the programme losing momentum. Additionally, an interview with a contact teacher revealed that one school had Ofsted inspections which led to de-prioritisation of GoActive with the school.

##### Staff Inconsistencies

For a few schools, teacher staffing was an issue for GoActive implementation. Merging of schools through Trusts meant that staff travelled between campuses. One contact teacher explained:

*The Year 9 tutors, that’s another, I guess, a barrier, in a way, is our Year 9 tutors. There’s only one form who has a consistent tutor throughout the week, all the other teachers have got at least two, perhaps three in some cases, of people coming in taking their register. So, you know, that’s an issue in school itself-that group of teachers, some of them might be teaching at [School 1], which is our other school up the road, in a morning, so they won’t be in [School 2], necessarily. So those people, who obviously have a bit more vested interest for these form groups, haven’t been around so much.*

(Contact Teacher, School F)

As such, the training provided to teaching staff may have only reached a minority of those who were involved. Additionally, staff turnover, or staff absence left supply (relief or substitute) teachers to facilitate running a programme they were unfamiliar with.

## 4. Discussion

This paper presents the mixed-methods process evaluation results of the GoActive programme. The results indicate that GoActive was implemented to some extent in all eight intervention schools; however, the complete GoActive programme was not implemented by any school. There was high variability in programme implementation within and between schools. At some schools, the mentor and/or in-class Year 9 peer leader roles were omitted. Reach was low; 37.9% of participants in intervention schools received the GoActive sessions. Despite this, qualitative data exploring dose received (satisfaction) was resoundingly positive, and quantitative data showed that 59.5% of Year 9 students thought that GoActive was fun. Mentors reported high satisfaction; 87.3% enjoyed participating in the programme. Recruitment and retention methods were positively received, with 76% of Year 9 students retained at 10 months post-intervention.

The absence of intervention effect on time spent in MVPA from participating in the GoActive intervention [14] may be due to inadequate implementation. The results indicate high variability in intervention fidelity for some elements, for example, the irregularity of GoActive sessions, mentor attendance, and implementation of in-class peer leaders. The latter of the results support our initial process evaluation findings regarding the implementation quality of the mentorship element [23]. The GoActive essential elements were linked to two facets of motivation informed by Self Determination Theory: extrinsic and intrinsic motivation [13,34]. Despite intervention components aligning with the basic needs for competence, autonomy, and relatedness, modifications or implementation issues potentially remove key underpinning principles of motivation, and subsequent behaviour change. Additionally, the considerable variance in the length of GoActive activities, due to school level factors (e.g., tutor time allocation, uniform requirements etc.) may suggest inconsistency in the amount of physical activity students engaged in across GoActive sessions.

The results presented in this paper together with previously published process evaluation data [23] demonstrate a lack of implementation for some key components, and discrepancies in reporting across dose delivered (completeness) across subgroups. The process evaluation showed variability in which specific components were implemented, and how these differed between school sites, and within schools (e.g., from tutor group to tutor group). In addition to the quantity of implementation, the quality of implementation between and within schools should also be considered. Despite quantitative reports indicating implementation of components such as in-class peer leaders, qualitative data shed light on the low quality of implementation of such components. The mixed-methods data suggest that the ambiguity of GoActive delivery roles, in particular the teacher and mentor role, may have been a primary factor impacting implementation quality, as opposed to the variation in facilitates as suggested for other physical activity interventions [35].

Despite the success of other peer-based physical activity interventions in increasing physical activity in adults, implementation of the mentorship aspects of GoActive was low, and did not demonstrate an impact on physical activity [36]. This finding is in line with the variable evidence of effectiveness of peer-delivered health promotion interventions for young people [37]. The GoActive findings indicate a need for continued mentor training regarding how to promote engagement in sessions, and targeted attention for participants showing decreasing participation early on. Additionally, from previous analysis, while some Year 9s thought that having in-class leaders would be a positive addition to the intervention, many Year 9s suggested they did not want to be in-class peer-leaders [23]. While current intervention design demonstrates enthusiasm for peer mentorship [38,39,40], our process evaluation demonstrates that there may be a mismatch in discourse around the assumption that peers tend to be greater influencers than parents or teachers, and whether adolescents want to be peer mentors/leaders.

In order for peer mentors to be effective, consideration should be given to the mentor selection process. For example, previous studies have used a peer nomination questionnaire to identify ‘influential’ peers to undertake mentor training, and provide support and encourage participation in the trial [41,42]. In addition, due to the reliance on mentor time, it may be best to build in mentorship into pre-existing leadership roles within the school. Noteworthy, the leadership role should be about wider participation, rather than a leadership role exclusively designed with a sports remit to avoid exacerbation of existing inequalities in schools.

Previous research has called for the design of multicomponent interventions to promote physical activity [7,43,44,45,46]. However, the multiple components included in the design of GoActive potentially hindered good implementation of the intervention. The elements that were implemented were potentially those considered the easiest to implement for schools. For example, mentorship was something most schools implemented, drawing on those who had previously been identified as mentors or leaders in the school. The low implementation of in-class peer leaders across all schools may have linked to the input required from mentors and teachers, and the lack of conceptualisation of their roles, but also the resistance from Year 9s expressed previously [23]. Additionally, programme elements that were under greater control of the participants, such as adding points to the GoActive website, and claiming rewards from the website, were inconsistent across schools, and between participants. On reflection, acknowledging the barriers continually faced in school-based work, simple, brief interventions may be preferable for schools. Work in this area is already underway [47,48].

The most commonly observed challenge to implementing GoActive was the understanding of mentor and teacher roles, followed by Year 9 student disengagement. Implementation difficulties may have also arisen from the provision of flexible intervention delivery, which was also identified in the Girls Active study [40]. Other challenges, including a lack of institutional support for contact teachers, time, and school level constraints, e.g., lack of school resources (space and time), are consistent with other research [18,49]. Compounding pressures on schools and educational systems requires physical activity researchers to do more to link health and educational outcomes. We affirm the importance of this recommendation from previous research [21]. Linking interventions to priorities and needs of the schools, for example, the National Curriculum Framework, or Education Inspection Framework, may establish greater importance of physical activity interventions, and justify the prioritisation of time to invest in health improvement interventions for their students. Curriculum-based approaches report high reach and dose of lessons taught [21,50]. Understanding the broader applicability of these lessons to out-of-school hours is the next challenge.

### 4.1. Wider Implications

The value of in-depth process evaluations should not be underestimated in the initial design of a study. Insight gained from the current process evaluation has prompted greater reflection regarding the implementation of complex interventions, and the consideration of using the school environment as a context for physical activity interventions. Our findings have a number of implications for the development and evaluation of public health improvement interventions for use in educational settings. Given the limited success of school-based physical activity promotion to date [17], we call for a step-change in our approach to intervention design and implementation.

The results presented here raise the question of the appropriateness and value of standardised intervention protocols across a multisite approach. The context of an intervention cannot be overlooked or undervalued. While the GoActive intervention itself was complex in nature, its interaction with its context was also highly complex. Our analysis highlights the importance of gaining both breadth, and depth in understanding of the context of individual schools. The disparity of resources, staffing, equipment and space between schools imposed clear challenges for the implementation of the intervention. While schools might provide significant reach to adolescents, there are multiple other factors that create challenges—for example, competing priorities, resource and time constraints, teacher/student rapport, or school culture around physical activity.

Co-production is seen as an inclusive method of intervention development, which may be thought to take into consideration contextual concerns [51]. The GoActive study demonstrates that, if we are to continue to embrace schools as an intervention setting, we need to do more than co-produce interventions; we need to understand each school’s culture, particularly as contexts change (e.g., moving to a Multi-Academy Trust (MAT) or an academy chain). We should endeavour to understand how a school’s culture can be a part of the intervention design or a complex intervention, for example, attitudes towards physical activity. An approach with greater emphasis on school empowerment through the selection of an appropriate and relevant programme to implement in their context, or implementing a protocol followed by input from schools to tailor the intervention could work best [21]. However, this more practice-based intervention may require new modes of evaluation other than randomised controlled trials.

In addition, researchers need to prioritise the selection of actionable and practically relevant implementation strategies and intervention mapping protocol to advance the quality of school-based interventions. A strong focus on implementation science in the initial design phase of school-based adolescent physical activity interventions is necessary. Evidence of embedded implementation adoption strategies within physical activity interventions is developing [52,53]. Following the PRACTIS guide steps, this comes under characterizing the parameters of the implementation setting, step 1 [54]. A stronger emphasis is required on the concepts of readiness, and resourcing [54], recognising the 10 domains that cover the core principles and methods of implementation science [55]. Additional consideration should also be given to the employment of Expert Recommendations for Implementing Change (ERIC), and School Implementation Strategies, Translating ERIC Resources (SISTER) to support selection and reporting of implementation strategies in schools [56].

### 4.2. Strengths and Limitations

By combining mixed-methods data on reach, recruitment, dose, and fidelity, the process evaluation provides detailed information regarding the implementation of a school-based physical activity intervention, compared to previous research. Although time consuming and researcher intensive, the diversity in data, including the perspectives of multiple subgroups, from all schools, allowed the triangulation of sources, an important factor to consider for interpreting our findings. Furthermore, data analysis for this process evaluation was undertaken prior to and independently from the analysis of the main trial outcomes [16].

However, this study has several limitations. The process evaluation focussed more on the quantity than quality of implementation. Implementation quality may be more important than fidelity and dose when impacting study outcomes [57], but it has been neglected in previous implementation evaluations of school-based physical activity interventions [58].

Participants identified as shy and inactive were purposefully sampled for interviews to provide a greater understanding of those most in need of health promotion engagement with whole school interventions. We were only able to interview two shy and inactive participants per school. This decision was made to enable the collection of breadth of data across all intervention schools to allow for contextual insights to be noted, as opposed to collecting in-depth data in a few schools. This allowed us to triangulate findings from subgroups to assess implementation fidelity across the school sites, as well as highlighting contextual factors. However, it prevented an in-depth exploration of this sub-population’s perspective on GoActive.

Evaluation of the training for intervention delivery, provided to facilitators, teachers and mentors, was not conducted. Observation or survey data collection on these dates would have provided insight into the consistency of what was delivered, the attendance at the sessions for each school, and the preparedness perspectives of those who were facilitating the programme.

Only a small number of teacher questionnaires were returned, and facilitator and mentor website logs were not completed consistently. This may lead to a biased evaluation, as those with strong views might have been more likely to provide their views. The team attempted to minimise this by creating a contact point within the school to act as a project champion, and by building rapport with teachers and facilitators via meetings and email. The views of teachers in school-based interventions is vital, particularly in terms of implementation. Barriers to this data collection need to be overcome in future research. Moreover, the lack of website logs mean that we were unable to assess how many GoActive sessions were conducted.

While the development of questions that were used to evaluate GoActive’s essential components provides specificity, a limitation of this approach is the lack of evidence for the reliability or validity of the scores that such scales generate. This is an issue that has broader applicability for other studies.

## 5. Conclusions

Multiple challenges and varying contextual considerations hindered the effective implementation of the GoActive programme to multiple school sites. The inability of the GoActive programme to elicit a positive change in MVPA compared to control may be attributed to the lack of implementation of GoActive components, explained by the ambiguity of the roles of teachers and mentors, Year 9 student disengagement, and school level constraints (e.g., teacher time, other priorities). This mixed-methods process evaluation provides important insight to understand the outcome results, and to guide future approaches to school-based physical activity intervention design and delivery. Recommendations for policy makers, future intervention design of school-based interventions, and researchers embarking upon upscaling of school-based interventions include a focus on implementation science, and deep consideration of contextual factors which may impact intervention scale-up. In-depth understanding of school culture, and innovative, potentially individualised, approaches to design may facilitate better implementation of school-based interventions. Our findings also suggest the need to consider alternative evaluation designs to account for contextual differences and diverse deliveries of an intervention across multiple sites.

## Figures and Tables

**Table 1:** GoActive essential elements.

GoActive Essential Elements	Descriptor
GoActive sessions	Each Year 9 class (tutor group) in the school chose two activities each week from a selection of 20 provided. Tutors were asked to deliver at least one GoActive session in tutor time per week.
Mentors	Mentors (older adolescents within the school) encouraged students to try these activities each week. It was recommended that schools employed 2 mentors per tutor group. Mentors were provided with Quick Cards (laminated print out resources) with activity instructions/tips.
	Mentors were asked to complete a log entry on the website about each of the GoActive sessions they ran.
In-class Year 9 leaders	It was recommended that tutor groups allocated two in-class Year 9 leaders (one male and one female), who changed weekly, to facilitate the sessions with mentors.
GoActive website use: points entered	Year 9s gained points for trying these new activities at any time in or out of school, and logged these on the password-protected GoActive intervention website. Points were gained every time students tried an activity; there was no expectation of time spent doing the activity as points were rewarded for taking part. Anonymised individual points were aggregated to facilitate class-level competition between tutor groups, displayed via school graphs showing leader boards within a school.
GoActive website use: website recorded claimed rewards	Year 9s received small rewards, such as a sports bag (10 points), t-shirt (20 points), or hoodie (50 points) for reaching individual points thresholds. They could claim rewards through the website. Mentors/tutors were tasked with approving the claimed reward, and distributing to Year 9s.

In addition to in-school leaders, a local authority-funded intervention facilitator, supported the programme during the first six weeks of delivery, and provided distant support thereafter. Further detail about the intervention is provided in supplementary file 1 (Table S1).

**Table 2:** Data collection and sampling.

Evaluation Method	Process Evaluation Outcome Addressed	Data Collection Timeframe	Participants	Number Completed	Response Rate
Questionnaire	Reach, recruitment, dose delivered (completeness), dose received (exposure), fidelity, dose received (satisfaction)	Mid-intervention (T2)	Year 9 students (intervention)	*n* = 1341	86.9% of 1543 baseline participants
Observation	Fidelity	During the first 6 weeks of the intervention/During the 12 week intervention Sessions ran Jan 2017-July, 2017	Form group, mentors, teachers in intervention schools	*n* = 8	8/8 intervention schools
Observation	Fidelity	During the last 6 weeks of the intervention Sessions ran April 2017–July 2017	Form group, mentors, teachers in intervention schools	*n* = 6	6/8 intervention schools
Individual interviews	Dose delivered (completeness), dose received (exposure), fidelity, dose received (satisfaction)		Year 9 students (intervention schools, identified as shy and inactive based on T1 baseline data questionnaire)	*n* = 16 (2 per school)	100%
Focus group interviews	Dose delivered (completeness), dose received (exposure), fidelity, dose received (satisfaction)		Year 9 students	*n* = 48	
			Mentors	*n* = 58	
Questionnaires	Reach, recruitment, dose delivered (completeness), dose received (exposure), fidelity, dose received (satisfaction)	Post-intervention (T3)	Year 9 students (intervention)	*n* = 1232	79.8% of baseline participants
			Year 9 form tutors	*n* = 20	*NA
			Mentors	*n =* 63	*NA
			Council facilitators	*n = 7*	100%
Individual interviews	Dose delivered (completeness), dose received (exposure), fidelity, dose received (satisfaction)		Council facilitators	*n* = 7	100%
Intervention delivery logs	Dose delivered (completeness), dose received (% of GoActive sessions received), fidelity		Mentors	*n* = 10	10/63 mentors provided partial data on intervention delivery logged on the GoActive website.
			Council facilitators	*n* = 7	5/7 facilitators provided partial data on intervention delivery logged on the GoActive website.
Website use	Dose delivered (completeness), fidelity, dose received (satisfaction)	From intervention start to 10-month follow-up (T4)	Year 9 students (intervention)	*n =* 714	46% of 1563 intervention participants

* Not available (NA) as denominator not known.

**Table 3:** GoActive process evaluation measures.

Process Evaluation Measure	Descriptor
Reach	Proportion of the intended priority audience that participates in the intervention [32]
Recruitment	Procedures used to approach and attract participants; including maintenance of participant involvement in intervention and measurement components of study [32,33]
Dose delivered (completeness)	Amount of units of each intervention component delivered [32]
Dose received (exposure)	Extent to which participants actively engage with, interact with, are receptive to the intervention; including initial and continued engagement [32]
Fidelity	Extent to which the intervention was implemented consistently as planned [16]
Dose received (satisfaction)	Participant (primary and secondary audiences) satisfaction with program [32]

**Table 4:** Dose delivered of GoActive essential components per school.

GoActive Intervention Essential Components		Implementation per School							
	School and *n* Participants	A (*n* = 140)	B (*n* = 169)	C (*n* = 207)	D (*n* = 229)	E (*n* = 232)	F (*n* = 116)	G (*n* = 219)	H (*n* = 231)
	School Level Socio-Economic Status	Low	Low	High	High	High	High	Low	Low
Baseline MVPA * (min/day) Mean (SD **)		34.3 (14.2)	33.9 (16.9)	39.1 (20.3)	38.2 (19.8)	32.3 (16.1)	37.4 (17.8)	37.4 (19.4)	33.3 (18.5)
Dose received-reported GoActive sessions at T3	% of Year 9s reporting at least one GoActive session in last two weeks.	21.3%	11.2%	49.5%	63.2%	47.9%	20.4%	55.4%	13.5%
Mentors	Number of mentors per school.	23	7	6	17	20	9	0	20
	N meetings recorded in the website log:	5	0	0	10	4	1	0	13
In-class Year 9 leaders	Percentage of Year 9s reporting having leaders in the class.	8.6%	10.0%	17.8%	54.6%	72.9%	30.2%	33.1%	27.1%
GoActive website use: points entered	Percentage recording points: Median (IQR ***) points recorded:	77.1% 8 (2–25)	8.9% 44 (17–58)	35.7% 12.5 (6–53)	60.7% 38 (5–43)	75.0% 58.5 (15–153)	19.8% 13 (4–39)	38.8% 24 (10–70)	41.6% 4 (2–4)
GoActive website use: rewards claimed ****	N rewards claimed via website:	11, by 7 students	15, by 8 students	28, by 16 students	58, by 28 students	230, by 106 students	13, by 7 students	50 by 22 students	3, by 1 student

* Moderate to vigorous physical activity (MVPA) measured at baseline with Axivity accelerometers. See Corder et al. (2020) [14] for more details on data collection. ** Standard deviation (SD). *** Interquartile range (IQR). **** 519 people recorded points but did not claim a reward via the GoActive website. An additional 606 rewards were issued but not logged via the website and cannot be matched with individual students.

**Table 5:** Summary of school implementation compared to GoActive essential elements planned as per the intended design of the intervention.

GoActive Essential Element	GoActive Tenet	School Implementation	
		Qualitative Data	Quantitative Data
GoActive sessions	Novelty; choice; flexibility	Reports from Year 9 students and older mentors reveal that there was limited time to discuss the choice of activity for the session. Choice and novelty were hindered due to a number of reasons: continuous choice of the same activity (often football), or the same ‘favourites’ were ‘picked for captains’ and they decided on the choice of activity. Very few Year 9s stated that they participated in a ‘novel’ activity. Year 9s did not engage with the choices provided, or had no desire to choose a novel activity. This resulted in mentors making the novel activity choice on behalf of the Year 9s.	Dose received of at least one GoActive class session in the last two weeks during the distant support phase ranged from 11.2% to 63.2% between schools, as reported by Year 9s (T3). 93.7% of the mentors and 84.2% of the teachers reported that GoActive sessions had been delivered at least once over the last two weeks (T3).
Mentors	Mentorship	Number of mentors varied based on number of tutor groups participating in GoActive, as well as school contact judgement on number of mentors applicable for the program. Evidence of mentorship was mixed from observations, and views from Year 9 focus groups: *The leaders and our form tutor don’t like encourage us to participate much or if they do, it’s like not very encouraging*. (Year 9 focus group, School D) *Just like, I don’t know, say if someone was sitting at the side they tried talking to them and getting them involved, like just trying to include everyone*.(Year 9 focus group, School C) Data from the observations indicated that some mentors modelled the behaviour, while others did not engage with the activity at all, aside from explaining rules and adjudicating. Year 9s heavily relied upon the mentor role, and attributed most of the interventions successes and failures to this role. Mentors did not meet the expectations of the Year 9s. Year 9s reported that QuickCard resources were seldom used.	8/9 schools had mentors. For the schools that had mentors, number of mentors ranged from 23 to 6.
In-class Year 9 peer leaders	Mentorship	Qualitative evidence suggests that very few tutor groups were able to implement in-class Year 9 peer leaders, if at all.	Quantitative data suggests that all schools implemented in-class Year 9 leaders in at least two GoActive sessions (ranging from 8.6–72.9% between schools.
GoActive website use: points logged	Competition	Year 9s discussed technical challenges to accessing the website, along with their inability to remember their password, or to sign into the website to log points, as key barriers.	46.5% (*n* = 717) of students in intervention schools assessed at baseline entered points via the GoActive website.
		Class-level competition, displayed via school graphs, was rarely referred to in individual and focus group interviews with all subgroups. One Year 9 focus group discussed being shown the graphs by their tutor. One mentor focus group revealed they had shown their tutor groups the graph. In an interview with a contact teacher, they described receiving the school graph by the GoActive facilitator, to which they discussed an intention of showing at an assembly. One facilitator mentioned the graphs when describing the tutor group participation in an individual interview.	No quantitative measure of school graph provision
GoActive website site: rewards claimed	Rewards	Year 9 students were informed of the GoActive reward system in a pre-intervention assembly held with every school. Reports of delayed reward distribution and confusion with where and whom to claim and collect rewards occurred. Rewards seemed to be collected by mentors and/or the GoActive contact teacher who presented these to students independently, rather than presenting them in class.	A total of 1014 rewards were claimed by at least 195 Year 9 students. Not all rewards were claimed through the website; 606 rewards could not be matched to individual students using website data.

## Supplementary Materials

The following are available online at https://www.mdpi.com/2227-9067/7/11/231/s1, Table S1: The TIDieR (Template for Intervention Description and Replication) Checklist*.
