# Supplementary material for: Reach, Recruitment, Dose, and Intervention Fidelity of the GoActive School-Based Physical Activity Intervention in the UK: A Mixed-Methods Process Evaluation
Source: Children (Basel). 2020 Nov 17;7(11):231. doi: 10.3390/children7110231 (PMC7698468; doi:10.3390/children7110231)
Supplement: Supplementary file 1 [file children-07-00231-s001.pdf]

Supplementary file 1: The TIDieR (Template for Intervention Description and Replication) Checklist*

TIDieR
Template for Intervention Description and Replication

Item number	Item	Where located ** Primary paper (page or appendix number)	Other † (details)
	**BRIEF NAME**		
**1.**	Get Others Active (GoActive)	Corder et al, 2015	______________
	**WHY**		
**2.**	Describe any rationale, theory, or goal of the elements essential to the intervention.<br><br>*Rationale*<br>Most adolescents are insufficiently active, and this inactivity tracks into adulthood, increasing the risk of diabetes, cancer and mortality. Pubertal, brain and social development during adolescence leads to new capacity for health behaviours, increasing the likelihood of long-term change.<br><br>There is a paucity of research which has focused on adolescents over the age of 13 years. The 2012 Chief Medical Officers report states the importance of physical activity among young people, and reports called for the provision of a more diverse and inclusive offer of physical activity within schools. These calls for the prioritisation of physical activity research highlight the lack of high quality research in this important group and an urgent need for the development and evaluation of potentially successful strategies.	Corder et al, 2015; Brown et al, 2017, p. 2	_____________

The GoActive physical activity promotion intervention was developed with substantial involvement from adolescents and was based on gaps in the existing evidence. Using an evidence-based iterative process involving the target group, we produced an intervention including components that were not necessarily commonly used in physical activity promotion, such as competition, rewards and mentorship. However, these components can be supported by evidence from promotion of other health behaviours. For example, various types of mentorship have been successfully used to improve sexual health behaviours, nutrition, substance abuse/use, and smoking, but cross-age mentoring to improve health behaviours is an understudied approach, especially in physical activity research.

Although GoActive was designed as a whole-population approach aiming to overcome stigmatization and potential detrimental mental health consequences of targeting particular subgroups in health promotion strategies, we wanted to ensure that the intervention was acceptable to those least likely to engage in physical activity. Therefore, the intervention was developed to include the opinions of those with characteristics that were deemed to be common in individuals who were hard to reach in PA promotions, including girls and those with low activity levels and high levels of shyness.

Theory

GoActive is broadly aligned with Self-Determination Theory (Ryan & Deci, 2000), however, our priority was to co-design the intervention with students and teachers. Therefore, we used theory flexibility to enable the incorporation of components strongly suggested in the development work, irrespective of whether they aligned with theory, such as rewards (Corder, Schiff, Kesten, & van Sluijs, 2015). Using elements of Self-determination Theory, GoActive aimed to increase physical activity through increased social support, self-efficacy, self-esteem, and friendship quality.

Goal

The primary aim of this study is to assess the 10-month effectiveness of the GoActive intervention to increase average daily objectively measured MVPA among 13–14-year-old adolescents. We will also assess the effect of GoActive immediately post intervention, and on the following secondary outcomes: (1) objectively assessed activity intensities during school time, weekday evenings and weekends; (2) student-reported physical activity participation, self-efficacy, peer support, social networks, self-esteem, friendship quality (proposed mediators) and well-being, and school level attendance and academic performance; and (3) body composition (body fat percentage and body mass index (BMI) z-score). We will investigate potential moderation of intervention effects by sex, socioeconomic status, ethnicity, baseline activity level and weight status, and potential mechanisms of effect by proposed mediators using a mixed-methods approach. Further, we will assess short-term (within-trial) and potential long-term cost-effectiveness of the GoActive intervention and will conduct a comprehensive process evaluation including questionnaires, focus groups, and individual interviews (with participants, mentors, teachers, and intervention facilitators), data from intervention logs and website analytics.

GoActive tenets

The six GoActive intervention tenets developed based on evidence and qualitative development work include: choice, novelty, mentorship, competition, rewards and flexibility. Each are described in more detail in Table 1.

Table 1: Six GoActive tenets

GoActive tenet	Detail
**Choice**	Relatively few physical activity promotion interventions specifically promote choices of physical activity. Interventions may either be ambiguous, for example, promoting physical activity in general rather than being specific about what behaviours to change or may organise specific activity sessions but do not provide a choice. However, qualitative evidence suggests that choice is vital for increasing adolescent physical activity (Rees et al., 2006). Autonomy for many behaviours increases during

	adolescence and therefore allowing adolescents input regarding intervention content is developmentally appropriate (Wilson et al., 2008). Giving adolescents a choice of activities may improve intrinsic motivation which is an important psychological factor for long-term maintenance of physical activity (Ryan & Deci, 2000; Wilson et al., 2008). Adolescents given an informed choice regarding their physical activity may also have better attendance in physical activity promotion programmes (Thompson & Wankel, 1980). Taken together this suggests that perceived choice and self-initiated behaviours may be instrumental for successful physical activity promotion (Wilson et al., 2008). Choice of activities was one of the most important factors identified by students in the GoActive Development Study.		
**Novelty**	GoActive aims to introduce adolescents to new activities and encourages students to earn points and rewards for trying these new activities. Self-determination theory suggests that novel, enjoyable and self-driven experiences will lead to more sustained behaviour change than external factors such as external reward or coercion (Ryan & Deci, 2000). GoActive provides discrete and achievable goals and timelines but gives participants flexibility to decide when, where and who to be active with. Although teachers, mentors and leaders will be encouraged to plan activities for their classes, individual students will be encouraged to plan when, where and with whom they will try the new activity. This technique is supported by evidence that if participants specify exactly when and where they plan to carry out a specified goal-driven action in advance they are more likely to achieve it (Roberts, Maddison, Magnusson, & Prapavessis, 2010).		
**Mentorship**	We aim to increase social networks, social support and self-efficacy for physical activity through mentorship. Our qualitative evidence suggests that older peer mentors and leaders are very appealing to adolescents to encourage physical activity promotion (GoActive Development Study). The tiered leadership structure of GoActive encourages older adolescents (mentors) and Year 9 elected peer-leaders to provide and facilitate physical activity opportunities for their class with structured support from researchers and teachers. Mentorship has been shown to have positive effects in obesity prevention/control interventions with some long term dietary improvements (Black et al., 2010). Mentorship programs have so far been relatively little researched regarding physical activity promotion (van Sluijs, Kriemler, &		

	McMinn, 2011). The introduction of peer-leaders and older adolescent mentors may encourage long term behaviour change by improving and extending participants' social networks. It is likely that any improvements to physical activity may last longer if grounded in an improved and extended social network (Macdonald-Wallis, Jago, Page, Brockman, & Thompson, 2011).
**Competition and rewards**	We also aim to encourage participation in new activities by utilising individual and class-level competitions with points and small gifts gained for trying new activities. Class and school-level activity challenges and competitions are successfully used in online physical activity promotion (www.gccjunior.org) and low-cost gifts improve participant engagement and retention in health promotion programmes (Tate, Larose, Espeland, & Wing, 2012).
**Flexibility**	Due to the nature of school-based work, we encouraged flexibility in the implementation of the intervention. Teachers were encouraged to use one of the five tutor times per week for activity. We also allowed the intervention to continue both in and out of school hours.

WHAT

3. Materials: Describe any physical or informational materials used in the intervention, including those provided to participants or used in intervention delivery or in training of intervention providers. Provide information on where the materials can be accessed (e.g. online appendix, URL). ____________

At a Year 9 assembly, each school involved in GoActive was shown a video introduction to GoActive, which can be found here: https://www.mrc-epid.cam.ac.uk/research/studies/goactive/about/. The intervention encouraged each Year 9 class (tutor group or home room class) choose two activities each week from a selection provided on the GoActive website. The GoActive website was housed at: http://www.goactive-uk.com/, but is no longer available for use. There were 20 activities available on the website, which encouraged using little or no equipment, and appealing to a wide variety of students (including Ultimate Frisbee, Zumba and Hula Hoop). Materials available on the password-protected GoActive intervention website include activity instructions (Quick Cards), which offered an overview of each activity, a short explanation, suggestions for adaptations and provide advice, safety tips and 'factoids', in addition to a short video introducing each activity. Quick cards were also printed for mentors and presented in-person.<br><br>Year 9 students also had an individual account on the GoActive website. Year 9 students gained points for trying these new activities at any time in or out of school. Points were gained every time they tried an activity; there was no expectation of time spent doing the activity as points are rewarded for the taking part itself. Individual students kept track of their own points privately on the study website and their points are entered into the between-class competition. Class rankings were available on the website to encourage teacher support and students receive small rewards (such as a sports bag, t-shirt, or hoodie) for reaching individual points thresholds.<br><br>Mentors, teachers and locally funded facilitators also had access to the GoActive website.<br><br>Locally funded facilitators received a 33-page manual. These materials were printed and provided to each facilitator. The manuals included an overview of the intervention, the background to the intervention, the process of delivery including the leadership structure, the roles of Year 9 peer leaders, mentors, teachers, and the facilitator; a greater focus on their role in the intervention including an overview of facilitator tasks, weekly	Brown et al, 2017, p. 2	

	school visits (first 6 weeks), launch assemblies, final assemblies, training teachers and mentors and dealing with inappropriate behaviour. The manual further clarifies the GoActive resources, specifically the QuickCards, videos, and website functionalities. The manual concludes with a description of the GoActive evaluation.		
**4.**	Procedures: Describe each of the procedures, activities, and/or processes used in the intervention, including any enabling or support activities.<br><br>*The GoActive intervention key components*<br>GoActive has four key intervention components: (1) GoActive sessions, (2) Older-year group mentors, (3) In-class Year 9 leaders, and the (4) GoActive website.<br><br>*The GoActive intervention*<br>Teachers were encouraged to use one tutor time (registration/roll call) weekly to do one of the chosen activities as a class, however, students gained points for trying these new activities in or out of school.<br><br>GoActive was implemented using a tiered-leadership system where mentors (older adolescents within the school) and peer-leaders (within each class) encouraged students to try GoActive activities each week. The mentors remain paired with each class for the duration of the intervention whereas the peer-leaders (two per class each week, one male and one female) changed every week.<br><br>The teachers, mentors and peer-leaders deliver the intervention with the support of a local council funded facilitator. 'Quickcards' provide information which allowed any of these individuals to lead the activities. For example, we suggest using YouTube for Zumba instruction as we want the students to be able to try activities without the barrier of needing a specific class. Similarly, we suggest doing these activities at home with a friend or relative to encourage out of school participation.	Brown et al, 2017, p. 2-3; Jong et al., 2020	____________

Although not based on one specific psychological theory, various behaviour change techniques were used in the programme (Table 2). These behaviour change techniques align with GoActive's six key tenets: Choice, Mentorship, Competition, Flexibility, Rewards and Novelty (Jong et al., 2018).

Table 2: Behaviour change techniques applied in the GoActive intervention

GoActive tenet	Behaviour Change Technique	Application in the GoActive intervention
Novelty, choice, mentorship	1.1 Goal setting (behaviour)	Tutor groups set a goal to try one new activity per week. Mentors encouraged Year 9 students to plan when and with whom they would try the activity
Competition	2.3 Self-monitoring of behaviour	Year 9 students record their participation in weekly new activities by entering points via the online website
Mentorship	3.1 Social support (unidentified)	Mentors, in-class Year 9 leaders, tutors and peers provided encouragement and support
	4.1 Instruction on how to perform behaviour	Quick Cards (laminated print out resources) and mentors provided activity instructions/tips
	6.1 Demonstration of the behaviour	Mentors were encouraged to model the behaviour; Quick Cards show examples of adolescents engaged in the behaviour
Competition	6.2 Social comparison	Points were awarded for trying activities. Anonymised individual points ranking allowed individual-level comparison; class-level competition were displayed via school graphs showing form group leader boards
Rewards	10.1 Material incentive (behaviour)	Year 9 students were informed of the GoActive reward system

	10.2 Material reward (behaviour)	Year 9 students were rewarded for obtaining points
	10.4 Social reward	Rewards were distributed in front of peers; Awards were presented at full year assembly at programme end
Mentorship	10.5 Social incentive	Year 9 students are informed that verbal praise will be provided
	12.2 Restructuring the social environment	A regular short (~20 minutes) intervention session is incorporated into the school timetable
	13.1 Identification of self as role model	Weekly elected Year 9 Peer Leaders act as role models; they support and encourage fellow students to try the chosen activities
Rewards	14.9 Reduce reward frequency	Year 9 students receive individual rewards on reaching point milestones (i.e. a sports bag (15 points), t-shirt (50 points), or hoodie (150 points)

WHO PROVIDED

5. For each category of intervention provider (e.g. psychologist, nursing assistant), describe their expertise, background and any specific training given. ______________

Facilitators: Local council funded facilitators are generally trained in health, health promotion and/or health education. All facilitators took part in a 1-day training session before the start of the GoActive intervention. Training was provided by members of the GoActive research team, and an external consultant specialised in adapting physical activity promotion to different contexts. Contents of the training focussed on providing background information on GoActive and its evaluation, a detailed overview of the intervention and the role of the facilitator in intervention delivery, the options for flexibility in intervention implementation, and the resources available to the facilitators and teachers/mentors. All facilitators additionally received a 33-page

Brown et al, 2017, p. 2-3; Jong et al., 2020

manual, and the research team was available throughout the intervention delivery phase if any questions or concerns arose.

Mentors: Mentors from participating intervention schools were provided with an after school training session for GoActive. At times this training was conducted with the form group tutors identified at the schools, and at other times this was conducted separately. The training included an overview of the intervention, a breakdown of their role, and details of their log ins for the GoActive website. Mentors were also provided with QuickCards as resources to support implementation. Mentors varied greatly in their experience in holding a prior mentor or leadership role.

Teachers: Year 9 form group tutors in participating intervention schools were provided with an after school training session for GoActive. At times this training was conducted with the mentors identified at the schools, and at other times this was conducted separately. Training included an overview of the intervention, a breakdown of their role, and details of their log ins for the GoActive website. Contact teachers (usually one per school, with one school having two contact teachers), sat in on the training provided to Year 9 form group tutors. Tutors and contact teachers covered a range of school subjects.

HOW

6. Describe the modes of delivery (e.g. face-to-face or by some other mechanism, such as internet or telephone) of the intervention and whether it was provided individually or in a group. Brown et al, 2017 ______________

Mode of delivery

The mode of contact teacher, mentor and facilitator training was face-to-face. This training occurred as a group: one group, day-long training session for facilitators, and one training session for mentors and teachers. Depending on the school, the teacher and mentor training either occurred together or separately. One

	facilitator dropped out from the intervention, and as such, additional one-to-one face-to-face facilitator training occurred for a facilitator who joined the intervention late.		
	The mode of intervention delivery was mixed, with GoActive sessions face-to-face led by mentors or peer-leaders, and supplementary points-based logging sessions occurring online via the GoActive website.		
	**WHERE**		
**7.**	Describe the type(s) of location(s) where the intervention occurred, including any necessary infrastructure or relevant features.		______________
	We implemented the GoActive intervention in eight secondary schools across Cambridgeshire and Essex, with a mixture of socioeconomic status, representative of UK variability.	Brown et al, 2017, p. 3	
	**WHEN and HOW MUCH**		
**8.**	Describe the number of times the intervention was delivered and over what period of time including the number of sessions, their schedule, and their duration, intensity or dose.		______________
	The intervention was delivered once in each school, between January 2017 through to July 2017. The anticipated number of sessions was 12, at least one GoActive session per week during form/tutor time. The duration varied per school due to variations of time scheduled for form time. Further sessions could be scheduled in addition to the 12, and participants were encouraged to, and rewarded for, activity outside of the GoActive sessions.	Corder et al., 2020, p. 4	
	**TAILORING**		

**9.**	If the intervention was planned to be personalised, titrated or adapted, then describe what, why, when, and how.<br><br>The intervention was not planned to be personalised, titrated or adapted per school context. However, the GoActive intervention was developed to be flexible to the needs of different schools.		______________
	**MODIFICATIONS**		
**10.**[ǂ]	If the intervention was modified during the course of the study, describe the changes (what, why, when, and how).<br><br>The intervention was briefly modified by the study team during the course of the study. Originally, it had been planned that the locally funded facilitators would conduct the mentor and teacher training. However, due to issues with recruitment and training facilitators, one of the study team delivered the training in schools to mentors and teachers.		______________
	**HOW WELL**		
**11.**	Planned: If intervention adherence or fidelity was assessed, describe how and by whom, and if any strategies were used to maintain or improve fidelity, describe them.	Jong et al., 2018	______________
	A process evaluation was designed to run alongside the GoActive outcome evaluation. The process evaluation was informed by the MRC guidance on the process evaluation of complex interventions. The main aim of the GoActive process evaluation was to understand what worked and why in the implementation of the GoActive	p. 3	

intervention, and to contribute to the interpretation of the findings of the effectiveness evaluation results. The objectives of the GoActive process evaluation are therefore:

1. To assess the reach, dose and fidelity of intervention delivery; to document how the intervention was implemented, and ascertain whether the intervention's essential functions (elements of the intervention) were adapted to suit individual settings
2. To explore the GoActive intervention from the perspective of Year 9 students, mentors, teachers, and facilitators, to describe participants' views of the intervention (including intervention acceptance)
3. To consider the maintenance and sustainability of the intervention and, if proven effective, the possible dissemination of the GoActive intervention

A trained qualitative researcher led on the process evaluation for GoActive. The process evaluation was designed to observe the implementation of GoActive and was not used to intervene with how a school implemented the GoActive intervention where deviations from the intervention protocol are detected.

Fidelity was assessed using a number of data collection methods, including observations, website analytics, interviews and questionnaires.

Qualitative data collection

Qualitative data was collected through observations, focus group and individual interviews, and mentor and facilitator written logs (housed on the GoActive website). All observations and interviews were carried out by the same trained mixed methods researcher. Reflection after each interview and discussion with independent researchers facilitated the development of the interview guides (e.g. to identify any required additions, or to pursue emerging themes),

p. 6

	Quantitative data collection<br>Quantitative data was collected through participant questionnaires, as well as via website analytics of points logged and pages visited within the GoActive website. A process evaluation section was included in the outcome questionnaires administered at T2 and T3 for all Year 9 students, both in the control and intervention arms of the trial. Separate process evaluation questionnaires were prepared for mentors, tutor group teachers in the intervention arm of the trial, and the local authority funded facilitators.	p. 5	
**12.[ǂ]**	Actual: If intervention adherence or fidelity was assessed, describe the extent to which the intervention was delivered as planned.<br><br>Please see Table 4, which indicates results from the process evaluation relevant to implementation fidelity.	Process evaluation paper forthcoming. Lead author: S Jong, p. 3	____________

References

Black, M. M., Hager, E. R., Le, K., Anliker, J., Arteaga, S. S., Diclemente, C., . . . Wang, Y. (2010). Challenge! Health promotion/obesity prevention mentorship model among urban, black adolescents. *Pediatrics, 126*(2), 280-288. doi:10.1542/peds.2009-1832

Corder, K., Schiff, A., Kesten, J. M., & van Sluijs, E. M. (2015). Development of a universal approach to increase physical activity among adolescents: the GoActive intervention. *BMJ Open, 5*(8), e008610. doi:10.1136/bmjopen-2015-008610

Jong, S. T., Brown, H. E., Croxson, C. H. D., Wilkinson, P., Corder, K. L., & van Sluijs, E. M. F. (2018). GoActive: a protocol for the mixed methods process evaluation of a school-based physical activity promotion programme for 13-14year old adolescents. *Trials, 19*(1), 282. doi:10.1186/s13063-018-2661-0

Macdonald-Wallis, K., Jago, R., Page, A. S., Brockman, R., & Thompson, J. L. (2011). School-based friendship networks and children's physical activity: A spatial analytical approach. *Social science & medicine, 73*(1), 6-12. doi:10.1016/j.socscimed.2011.04.018

Rees, R., Kavanagh, J., Harden, A., Shepherd, J., Brunton, G., Oliver, S., & Oakley, A. (2006). Young people and physical activity: a systematic review matching their views to effective interventions. *Health Education Research, 21*(6), 806-825. doi:10.1093/her/cyl120

Roberts, V., Maddison, R., Magnusson, J., & Prapavessis, H. (2010). Adolescent physical activity: does implementation intention have a role? *Journal of Physical Activity & Health, 7*(4), 497-507. Retrieved from http://www.ncbi.nlm.nih.gov/pubmed/20683092

Ryan, D., & Deci, E. (2000). Self-determination theory and the facilitation of intrinsic motivation, social development, and well-being. *American Psychologist, 55*, 68-78.

TIDieR checklist

Tate, D., Larose, J., Espeland, M., & Wing, R. (2012). Study of Novel Approaches to Prevention (SNAP) of Weight Gain in Young Adults: Rationale, Design and Development of Interventions. *ISBNPA Annual Meeting*.

Thompson, C., & Wankel, L. (1980). The effect of perceived activity choice upon frequency of exercise behavior. *J Appl Soc Psychol, 10*, 436-443.

van Sluijs, E. M., Kriemler, S., & McMinn, A. M. (2011). The effect of community and family interventions on young people's physical activity levels: a review of reviews and updated systematic review. *British Journal of Sports Medicine, 45*(11), 914-922. doi:10.1136/bjsports-2011-090187

Wilson, D. K., Kitzman-Ulrich, H., Williams, J. E., Saunders, R., Griffin, S., Pate, R., . . . Sisson, S. B. (2008). An overview of "The Active by Choice Today" (ACT) trial for increasing physical activity. *Contemporary clinical trials, 29*(1), 21-31. doi:10.1016/j.cct.2007.07.001
